# Probiotics fortify intestinal barrier function: a systematic review and meta-analysis of randomized trials

**DOI:** 10.3389/fimmu.2023.1143548

**Published:** 2023-04-24

**Authors:** Yanfei Zheng, Zengliang Zhang, Ping Tang, Yuqi Wu, Anqi Zhang, Delong Li, Chong-Zhi Wang, Jin-Yi Wan, Haiqiang Yao, Chun-Su Yuan

**Affiliations:** ^1^ School of Traditional Chinese Medicine, Beijing University of Chinese Medicine, Beijing, China; ^2^ National Institute of Traditional Chinese Medicine (TCM) Constitution and Preventive Medicine, Beijing University of Chinese Medicine, Beijing, China; ^3^ Traditional Chinese Medicine College, Inner Mongolia Medical University, Inner Mongolia, China; ^4^ Tang Center for Herbal Medicine Research, The University of Chicago, Chicago, IL, United States; ^5^ Department of Anesthesia and Critical Care, The University of Chicago, Chicago, IL, United States

**Keywords:** intestinal barrier function, randomized controlled trial, immune function, inflammation, probiotics

## Abstract

**Background:**

Probiotics play a vital role in treating immune and inflammatory diseases by improving intestinal barrier function; however, a comprehensive evaluation is missing. The present study aimed to explore the impact of probiotics on the intestinal barrier and related immune function, inflammation, and microbiota composition. A systematic review and meta-analyses were conducted.

**Methods:**

Four major databases (PubMed, Science Citation Index Expanded, CENTRAL, and Embase) were thoroughly searched. Weighted mean differences were calculated for continuous outcomes with corresponding 95% confidence intervals (CIs), heterogeneity among studies was evaluated utilizing I2 statistic (Chi-Square test), and data were pooled using random effects meta-analyses.

**Results:**

Meta-analysis of data from a total of 26 RCTs (n = 1891) indicated that probiotics significantly improved gut barrier function measured by levels of TER (MD, 5.27, 95% CI, 3.82 to 6.72, P < 0.00001), serum zonulin (SMD, -1.58, 95% CI, -2.49 to -0.66, P = 0.0007), endotoxin (SMD, -3.20, 95% CI, -5.41 to -0.98, P = 0.005), and LPS (SMD, -0.47, 95% CI, -0.85 to -0.09, P = 0.02). Furthermore, probiotic groups demonstrated better efficacy over control groups in reducing inflammatory factors, including CRP, TNF-α, and IL-6. Probiotics can also modulate the gut microbiota structure by boosting the enrichment of Bifidobacterium and Lactobacillus.

**Conclusion:**

The present work revealed that probiotics could improve intestinal barrier function, and alleviate inflammation and microbial dysbiosis. Further high-quality RCTs are warranted to achieve a more definitive conclusion.

**Clinical trial registration:**

https://www.crd.york.ac.uk/PROSPERO/display_record.php?RecordID=281822, identifier CRD42021281822.

## 1 Introduction

Intestinal barrier function is closely related to the pathogenesis of various immune and inflammatory diseases (1–3). The intestinal barrier, including surface mucus, epithelial layer, and immune defense, is a dynamic entity interacting with and responding to a variety of stimuli (2). The physical barrier has epithelial and mucus components tightly linked to different cellular junctions, including desmosomes, adherens junctions, and tight junctions (4). The primary function of the intestinal epithelium is to act as a barrier that limits the interaction between luminal contents, such as gut bacteria, the underlying immune system, and the rest of the body (5). Moreover, the biological barrier mainly comprises the normal intestinal flora and can regulate the intestinal microecological balance (6). A leaky gut occurs due to the perturbation of gut barrier homeostasis with increased epithelial permeability and perhaps microbial dysbiosis, which can lead to the passage of toxins, antigens, and bacteria from the lumen to enter the bloodstream, thus resulting in diverse systemic consequences, including increased inflammation, oxidative stress, and blunted insulin sensitivity (1, 7, 8).

The intestinal microbiota plays an essential role in maintaining gut homeostasis and functionality in the presence of pro-inflammatory and anti-inflammatory microbes. Intestinal commensal microbes promote health, in part, by reinforcing the gut barrier *via* direct and indirect mechanisms (9). Probiotics are living microorganisms that confer health benefits to the host when given proper amounts and durations (10). Probiotics and intestinal symbionts can modulate the intestinal barrier function of the host through their surface molecules and metabolites (11). Thus, probiotics could restore intestinal health by attenuating inflammation and strengthening the epithelial barrier. Therefore, probiotics may be essential in treating diseases by improving intestinal barrier function.

A prior meta-analysis suggested that supplementation with probiotics can be beneficial in protecting the gut mucosal barrier in patients with colorectal cancer after an operation (12). However, an exhaustive assessment of probiotics regulating intestinal barrier function in multiple disease conditions is still missing. This study aims to comprehensively evaluate the role of probiotics in contributing to intestinal barrier function, and the related immune function, inflammatory status, and gut microbiota composition, thus providing a better understanding of the beneficial effects of probiotic supplementation.

## 2 Methods

### 2.1 Study protocol

This systematic review and meta-analysis was reported following the Preferred Reporting Items for Systematic Reviews and Meta-Analysis (PRISMA) (13), and the PRISMA checklist is available in **Supplementary Table S1**. The protocol for the present study has already been registered at PROSPERO (No. CRD42021281822).

### 2.2 Data source and search strategy

The data source of this review was gained by searching four major biomedical databases: PubMed, Web of Science: Science Citation Index Expanded, Cochrane Central Register of Controlled Trials (CENTRAL), and Embase from inception to February 10, 2022. Keywords in the search strategy included probiotics, gut barrier, intestinal barrier, leaky gut, random clinical trials, etc. No language restrictions or limitations of the published year were imposed. The detailed search strategies were provided in **Supplementary Materials** (**Tables S2–S5**). All the search results from four different databases were stored in EndNote Library 20 for manageable convenience.

### 2.3 Study selection criteria

All articles are irrespective of population. Two reviewers independently screened the titles and abstracts and subsequently assessed the eligibility of the full texts of identified studies to select potentially eligible studies. Disagreements were resolved by a third reviewer if there were still discrepancies after the discussion between the two reviewers.

The inclusion criteria for randomized controlled trials (RCTs) that needed to be met were as follows: the intervention group should add single- or multiple-strain probiotics, and outcomes were at least one parameter of intestinal barrier function assessed. We excluded RCTs that interventions were not probiotics or whose outcomes were not relevant to intestinal permeability. Review articles, meta-analysis articles, and animal studies were omitted. However, the references of these publications were screened for potentially includable studies.

### 2.4 Data extraction

Vital data information about each study, including the author’s name, published year, country, type of study, characteristics of the study population (sex, age, body weight, etc.), experimental design, duration of intervention, sample size; and outcomes of the intestinal barrier function, gut microbiota, inflammatory indicators, and immune functions were extracted from each article that fulfilled the inclusion criteria. Two reviewers carried out this work independently and resolved disagreements by consensus.

### 2.5 Risk of bias assessment

Two reviewers conducted the risk of bias assessment independently, and different opinions were sent to another senior author to be solved. We applied the Cochrane Collaboration’s risk of bias through the software Review Manager (RevMan 5.4) to evaluate the quality of the studies included in our system review (14). There are six evaluation items in different aspects, including the selection bias of random sequence generation as well as allocation concealment, the performance bias of blinding of participants and personnel, the detection bias of blinding of outcome assessment, the attrition bias of incomplete outcome data, the reporting bias of selective reporting and other bias, which were judged as the conclusion of low, high, or unclear risk.

### 2.6 Data synthesis and analysis

The meta-analysis was performed using the Review Manager software (RevMan 5.4) (15). We calculated weighted mean differences for continuous outcomes with corresponding 95% confidence intervals (CIs). In the data analysis of included studies, a mean difference (MD) model in RevMan will be applied if the data units are uniform; otherwise, a standard mean difference (SMD) model will be employed. If more than one time point of outcome during the treatment were reported, the data from the last time point would be extracted for pooled analysis. Heterogeneity among studies was evaluated utilizing *I* ^2^ statistic (Chi-Square test). A fixed-effect model was applied for the analysis of homogenous data (*I* ^2^ < 50%); alternatively, a random-effect model will be applied for heterogeneous data (16). Funnel plot asymmetry was examined to evaluate the publication bias. Subgroup analyses were performed based on country, population characteristics, age, study design, treatment duration, etc., to explore possible causes of heterogeneity among each result indicator.

All data pooling in the meta-analysis was in the form of mean ± standard deviation (SD). When the original data were reported as median and range (or interquartile range), we estimated the mean and SD using an online calculator provided by Luo et al. (17). Engauge Digitizer software (version 11.1) was applied to extract data from the studies which provide data in figures other than numerically.

### 2.7 Subgroup analyses

Subgroup analysis was conducted based on the main possible contributing factors that may cause heterogeneity, including country, population characteristics, and duration of treatment.

## 3 Results

### 3.1 Study selection

**Figure 1:**
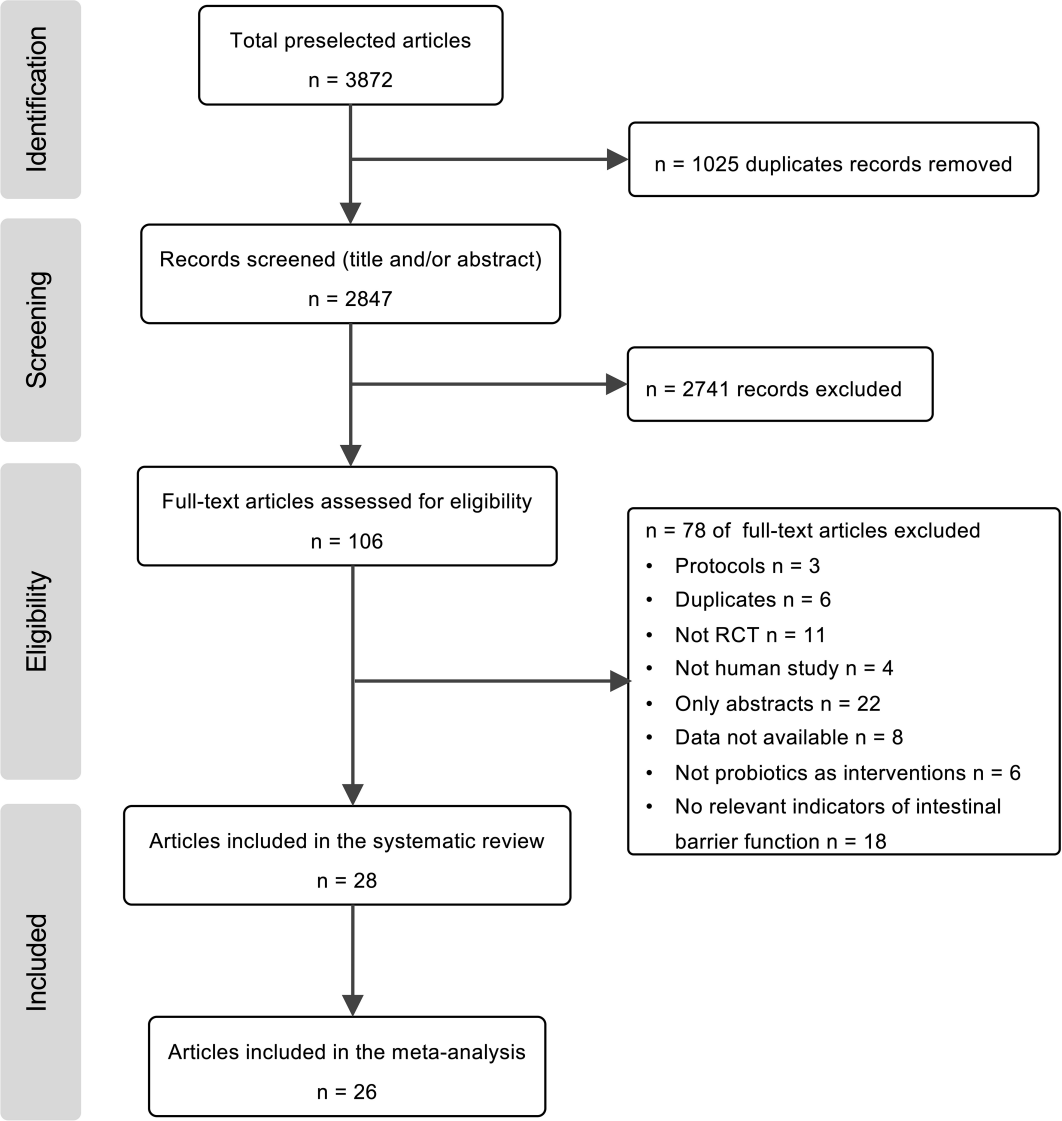
Flow diagram of study selection progress for the systematic review and meta-analyses.

A total of 3872 potentially relevant studies were retrieved after a combined search from four databases, and 2847 remaindered to go through the articles’ titles and abstracts after excluding the duplicates. The number of articles was reduced to 106 for further full-text screening, 28 articles were included in the qualitative synthesis, and 26 RCTs were included in the meta-analysis (**Figure 1**).

### 3.2 Characteristics of included studies

The characteristics of the 28 included studies were all RCTs (**Table 1**). A total of 1891 randomized participants divided into probiotic intervention groups (n = 955) and control (n = 936) groups were included in this systematic review. Across the included studies, the study population mainly combined two kinds of people, patients and athletes. The characteristics of patients are mainly related to hepatic diseases (23, 24), acute pancreatitis (19, 31, 33), gastrointestinal diseases (20, 21, 27–29, 35, 36, 42–45), metabolic disorders (26, 39), and acute diseases (18, 30, 32, 41). Apart from those kinds of patients, there were also RCTs on migraine (22), early sepsis (38), and psychological stress (37). Studies of probiotics on healthy subjects, e.g., endurance-trained men (25), male runners (34), and division I male baseball athletes (40) are also included. All included articles were published between 2005 and 2021, and six were published in the last three years.

**Table 1:** Characteristics of included randomized controlled trials.

Study	Country	Design	Population characteristics	Age	Control	Probiotics	Study duration	n	Intestinal barrier function	Gut microbiota	Other indicators
18	Canada	Randomized, double blind, placebo-controlled trial	Critically ill patients	Control: 64.9 ± 16.9 Probiotics: 60.4 ± 17.9	Placebo	VSL#3: Lactobacillus (L. casei, L. plantarum, L. acidophilus, and L. delbrueckii subsp. Bulgaricus), Bifidobacterium (B. longum, B. breve, and B. infantis) and Streptococcus salivarius subsp. Thermophilus (2 sachets twice daily)	7 days	Control: 9 Intervention: 10	L/M	—	CRP, IgA, IgG
19	Netherlands	Randomized, placebo-controlled, double-blind, multicenter trial	Patients with a first episode of predicted severe acute pancreatitis	60.5 ± 16.0	Placebo	Ecologic 641: a mixture of 6 lactobacillus, lactococcus, or bifidobacteriae	7 days	Control: 144 Intervention: 152	IFABP, PEGs, NOx	—	—
20	Italy	Crossover randomized double-blind controlled trial	Patients with irritable bowel syndrome	48 ± 11	Placebo (maltodextrins, corn starch, silicon dioxide)	LBB: Bifidobacterium longum BB536 and Lactobacillus rhamnosus HN001 with vitamin B6 (1 sachet pack daily)	60 days	Control: 25 Intervention: 25	L/M, sucralose recovery	—	—
21	China	Randomized, parallel-group, controlled trial	Patients with colorectal cancer	Control: 59.8 ± 18.7 Probiotics: 60.3 ± 17.2	Placebo	Combined Clostridium Butyricum and Bifidobacterium Capsules,Live (capsule: 3 capsules thrice daily)	12 days	Control: 35 Intervention: 35	TER, Mannitol permeability	Bifidobacteria, Lactobacilli, Enterobacterium	—
22	Netherlands	Randomized placebo-controlled study	Patients with migraine	Control: 38 (18–70) Probiotics: 42 (18–69)	Placebo (2g of the carrier of the probiotic product; maize starch and maltodextrin powder)	Bifidobacterium bifidum W23, B. lactis W52, Lactobacillus acidophilus W37, Lactob. brevis W63, Lactob. casei W56, Lactob. salivarius W24, Lactococcus lactis W19 and Lactoc. lactis W58 (2g sachets once daily)	12 weeks	Control: 29 Intervention: 31	L/M, Zonulin in feces and serum	—	CRP, IL-6, IL-10, TNF-α
23	Austria	Randomized, double blind, placebo-controlled study	Patients with cirrhosis	Control: 56 (50; 63) Probiotics: 60 (54; 64)	Placebo	Bifidobacterium bifidum W23, Bifidobacterium lactis W52, Lactobacillus acidophilus W37, Lactobacillus brevis W63, Lactobacillus casei W56, Lactobacillus salivarius W24, Lactococcus lactis W19 and Lactococcus lactis W58 (6g daily)	6 months	Control: 36 Intervention: 44	L/M, DAO, ET Zonulin in fecal, sucralose recovery	—	CRP
24	Republic of Korea	Randomized, double blind, placebo-controlled study	Patients with chronic liver disease	Control: 53.3 ± 9.8 Probiotics: 54.4 ± 8.4	Placebo	Bifidobacterium bifidum, Bifidobacterium lactis, Bifidobacterium longum, Lactobacillus acidophilus, Lactobacillus rhamnosus, and Streptococcus thermophilus (capsule: twice daily)	4 weeks	Control: 25 Intervention: 25	L/M	Bifido group, Lacto group	—
25	Austria	Randomized, double-blinded, placebo-controlled trial	Endurance trained men	Control: 38.2 ± 4.4 Probiotics: 37.6 ± 4.7	Placebo (a matrix cornstarch, maltodextrin, vegetable protein)	Bifidobacterium bifidum W23, Bifidobacterium lactis W51, Enterococcus faecium W54, Lactobacillus acidophilus W22, Lactobacillus brevis W63, and Lactococcus lactis W58 (4g daily)	14 weeks	Control: 12 Intervention: 11	Zonulin in fecal	—	IL-6, TNF-α
26	Austria	An open label, randomized pilot study	Patients with metabolic syndrome	Control: 54.5 ± 8.9 Probiotics: 51.5 ± 11.4	Standard treatment	YAKULT light: L. casei Shirota (liquid: 3 bottles of 65 ml daily)	3 months	Control: 15 Intervention: 13	Recovery of saccharose, L/M, DAO	—	—
27	China	Double-center and double-blind randomized clinical trial	Patients with colorectal liver metastases	Control: 60.16 ± 16.20 Probiotics: 65.62 ± 18.18	Placebo (maltodextrin)	Lactobacillus plantarum, Lactobacillus acidophilus-11 and Bifidobacterium longum-88 (capsules: 2g daily)	16 days	Control: 58 Intervention: 59	ET, Zonulin in serum	—	—
28	China	Randomized, double-blind, placebo-controlled, prospective study	Patients undergoing elective colorectal surgery	Control: 65.7 ± 9.9 Probiotics: 65.3 ± 11.0	Placebo (maltodextrin)	Lactobacillus plantarum, Lactobacillus acidophilus-11 and Bifidobacterium longum-88 (capsules: 2g daily)	16 days	Control: 50 Intervention: 50	L/M, TER, I-FABP	Bifidobacterium, Lactobacillus, Enterobacteriaceae	—
29	China	Double-center and double-blind randomized clinical trial	Patients with colorectal cancer	Control: 62.28 ± 12.41 Probiotics: 66.06 ± 11.02	Placebo (maltodextrin)	Lactobacillus plantarum, Lactobacillus acidophilus-11 and Bifidobacterium longum-88 (capsules: 2g daily)	16 days	Control: 75 Intervention: 75	L/M, serum zonulin, TER	—	—
30	UK	A prospective randomized trial	Critically ill patients.	Control; 71 (28-87) Probiotics: 71 (28-90)	Conventional therapy	ProViva: L. plantarum 299v (liquid: 500 ml daily)	15 days	Control: 51 Intervention: 52	L/R	—	IL-6
31	China	Prospective, randomized, single-blinded, parallel design clinical trial	Patients with acute pancreatitis	Control: 58.4 ± 19.1 Probiotics: 54.3 ± 13.1	Parenteral nutrition	Lactobacillus plantarum (liquid: 100ml daily through the nasojejunal tube)	1 week	Control: 38 Intervention: 36	L/R	Bifidobacteria, Lactobacteria, Enterococci	CRP
32	Iran	Randomized, double−blind, placebo−controlled trial	Critically ill patients	Control: 35.60 ± 5.03 Probiotics: 33.60 ± 5.50	Placebo	VSL#3: Lactobacillus casei, Lactobacillus plantarum, Lactobacillus acidophilus, Lactobacillus delbrueckii subsp., Bifidobacterium longum, Bifidobacterium breve, and Bifidobacterium infantis and Streptococcus salivarius subsp. Thermophilus. (2 sachets daily)	7 days	Control: 20 Intervention: 20	—	—	IL−6
33	India	Randomized, double−blind, placebo−controlled trial	Patients with acute pancreatitis	Control: 40.19 ± 17.43 Probiotics: 41 ± 20.72	Placebo	Lactobacillus acidophilus, Bifidobacterium longus, Bifidobacterium bifidum, and Bifidobacterium infantalis (4 sachets daily)	7 days	Control: 26 Intervention: 24	L/M	—	hsCRP, IgG, IgM
34	Australia	Double-blind, placebo-controlled cross-over trial	Male runners	27 ± 2	Placebo (skim milk powder)	Lactobacillus acidophilus, L. rhamnosus, L. casei, L. plantarum, L. fermentum, Bifidobacterium lactis, B. breve, B. bifidum and Streptococcus thermophilus (capsule: 1 capsule daily)	4 weeks	Control: 10 Intervention: 10	L/R, LPS	—	IgM, IL-6, IL-10, TNF-α
35	India	Randomized, double-blind, placebo-controlled trial	Children with gastroenteritis	6 months -5 years	Placebo	Lactobacillus rhamnosus GG (1 capsule given once daily in boiled and cooled milk)	4 weeks	Control: 59 Intervention: 65	L/M	—	—
36	Canada	Randomized, double-blind, placebo-controlled study	Patients with untreated celiac disease	Control; 40 (20-71) Probiotics: 46 (29-62)	Placebo (rice flour, dehydrated potato powder, cellulose powder, and hydroxypropyl-methylcellulose)	Bifidobacterium infantis natren life start strain super strain (capsule: 2 capsules thrice daily)	3 weeks	Control: 10 Intervention: 12	L/M	—	IL-6
37	Italy	Randomized, double-blind, placebo-controlled, cross-over trial	Healthy adults who self-reported psychological stress	20-35	Placebo (liquid mixture)	Lactoflorene^®^ Plus: Lactobacillus acidophilus LA-5^®^, Bifidobacterium animalis subsp. lactis, BB-12^®^, Lactobacillus paracasei subsp. paracasei, L. CASEI431^®^, Bacillus coagulans BC513, zinc and B vitamins (niacin, B1, B2, B5, B6, B12 and folic acid) (liquid: two 10ml bottles daily)	45 days	Control: 25 Intervention: 25	—	—	IgA, IL-10, TNF-α
38	Austria	Randomized, double blind, placebo-controlled pilot study	Patients with early sepsis	54 (47; 60)	Placebo	Lactobacillus plantarum W1, Lactobacillus paracasei W20, Bifidobacterium bifidum W23, Lactobacillus salivarius W24, Lactobacillus acidophilus W37, Bifidobacterium lactis W51, Enterococcus faecium W54, Lactobacillus acidophilus W55, Lactobacillus plantarum W62, Lactobacillus rhamnosus W71 (5g twice daily)	28 days	Control: 4 Intervention: 5	DAO, ET, Zonulin in stool	—	—
39	Thailand	Randomized, double-blind, placebo-controlled study	Patients with type 2 diabetes mellitus	Control: 61.78 ± 7.73 Probiotics: 63.50 ± 5.94	Placebo (corn starch)	L. paracasei HII01 (50 × 109 CFU/day)	12 weeks	Control: 18 Intervention: 18	ZO-1, LPS	—	hsCRP, IgA, IL-6, IL-10, TNF-α
40	USA	Randomized, double blind, placebo-controlled study	Division I male baseball athletes	20.1 ± 1.5	Placebo (maltodextrin)	Bacillus subtilis DE111 (capsule: 1.2 billion CFU/capsule)	12 weeks	Control: 12 Intervention: 13	Zonulin in serum	—	IL-10, TNF-α
41	China	Single-blind, randomized controlled trial	Critically ill patients.	Control: 81 (61; 95) Probiotics: 81 (70; 96)	Placebo	Clostridium butyricum (tablet: 1 sachet thrice daily)	14 days	Control: 33 Intervention: 27	DAO, LPS	—	IL-10, TNF-α
42	Netherlands	Randomized, double blind, placebo-controlled study	Patients with ulcerative colitis	Control: 51.1 ± 11.9 Probiotics: 51.8 ± 13.3	Placebo (maize starch and maltodextrins)	Ecologic^®^ 825: Bifidobacterium bifidum W23, Bifidobacterium lactis W51, Bifidobacterium lactis W52, Lactobacillus acidophilus W22, Lactobacillus casei W56, Lactobacillus paracasei W20, Lactobacillus plantarum W62, Lactobacillus salivarius W24, and Lactococcus lactis W19 (2 sachets daily of 3g)	12 weeks	Control: 12 Intervention: 13	L/R, S/E, Zonulin in serum and faecal	—	IL-6, IL-10, TNF-α
43	China	Randomized, parallel-group, controlled trial	Patients undergo colonic surgery	67.3 (37–82)	Preoperative bowel preparation methods	Lactobacillus acidophilus LA11 (granule; 2g daily)	≥5 days	Control: 30 Intervention: 30	—	—	IgA
44	China	Single-center prospective randomized control study	Patients with colorectal cancer	Control: 61.5 (46.0–82.0) Probiotics: 67.5 (45.0–87.0)	Placebo (maltodextrins)	B longum, L acidophilus and Enterococcus faecalis (capsule: 3 capsules thrice daily)	3 days	Control: 30 Intervention: 30	D-LA, ET	—	CRP, IgA, IgG, IgM, IL-6
45	China	Randomized, parallel-group, controlled trial	Patients with diarrhea secondary to leukemia chemotherapy	Control: 9.26 ± 1.84 Probiotics: 9.17 ± 1.92	Routine symptomatic support	Bifidobacterium longum, Lactobacillus acidophilus and Enterococcus faecalis (capsule: 1 capsule twice daily)	2 weeks	Control: 45 Intervention: 45	DAO, D-LA, ET	Bifidobacterium, Lactobacillus, Enterococcus, Enterobacteriaceae	IL-6, TNF-α

"__" means not mentioned.

In most included studies, *Bifidobacterium* and *Lactobacillus* were added to evaluate the efficacy of probiotics compared to control groups. Probiotics were administered in different dosage forms, including capsules, tablets, and liquids. The duration of the intervention varied from 3 days to 6 months.

Outcomes were mainly measured in four aspects: intestinal barrier function indicators, inflammatory factors, immune function indicators, and gut microbiota structures. Evaluation of intestinal barrier function has different indicators, including the levels of diamine oxidase (DAO), D-lactic acid (D-LA), ratios of lactulose to mannitol (L/M), and lactulose to rhamnose (L/R), endotoxin (ET), lipopolysaccharide (LPS), serum and fecal zonulin, intestinal fatty acid-binding protein (I-FABP), and transepithelial resistance (TER). Inflammatory factors of tumor necrosis factor-alpha (TNF-α), interleukin 6 (IL-6), interleukin 10 (IL-10), C-reactive protein (CPR), and high-sensitivity C-reactive protein (hsCRP) were measured to assess the anti-inflammatory function of probiotics supplementary. Moreover, Immunoglobulin A (IgA), Immunoglobulin G (IgG), and Immunoglobulin M (IgM) levels were measured to assess whether probiotics could improve immune function.

### 3.3 Quality of included studies

All the included studies were assessed for quality in different aspects using the Cochrane Collaboration tool (**Figures S1**; **S2**). Among the 28 RCTs, random sequence generation in 19 studies tended to be a low-risk bias, and nine did not report the method of random sequence generation. In allocation concealment, 16 studies showed low bias, and 12 did not provide information on allocation concealment. Eleven studies provided no information on the blinding of participants and personnel, and 24 did not report the blinding of outcome assessment. Ten studies did not provide details on incomplete outcome data, and 26 RCTs did not provide enough information to evaluate selective reporting. The most common risks of other biases in these studies were assessment biases and possibly a lack of adequate control of various factors, such as doses of probiotics, lengths of treatment, and methods of assessments.

### 3.4 Effects of probiotics on intestinal barrier function

Different kinds of methods were applied to assess the intestinal barrier function. In the 28 studies, a total of 19 test methods were used; seven assessed intestinal permeability, six assessed intestinal integrity, one related to bacterial translocation, three related to harmful factors, and two other indicators (**Table 2**).

**Table 2:** Methods applied to assess the intestinal barrier function and the efficacy of probiotics.

Methods	Number	Studies	Participants	Mean difference (95% CI)	*P* - value	Heterogeneity
L/M	8	18, 22, 26, 28, 29, 33, 35, 36	517	-0.02 [-0.03, 0.00]	*P* = 0.06	*I^2 = ^* 87%
Fecal Zonulin	5	22, 23, 25, 38, 42	190	-1.63 [-14.06, 10.81]	*P* = 0.80	*I^2 = ^* 50%
Serum Zonulin	5	22, 27, 29, 40, 42	385	-1.58 [-2.49, -0.66]	*P* = 0.0007	*I^2 = ^* 92%
ET	4	27, 38, 44, 45	219	-3.20 [-5.41, -0.98]	*P* = 0.005	*I^2 = ^* 97%
DAO	5	23, 26, 38, 41, 45	268	-0.31 [-1.03, 0.40]	*P* = 0.39	*I^2 = ^* 86%
L/R	4	30, 31, 34, 42	143	-0.04 [-0.13, 0.04]	*P* = 0.33	*I^2 = ^* 97%
TER	3	21, 28, 29	170	5.27 [3.82, 6.72]	*P* < 0.00001	*I^2 = ^* 0%
LPS	3	34, 39, 41	113	-0.47 [-0.85, -0.09]	*P* = 0.02	*I^2 = ^* 48%
D-LA	2	44, 45	150	-1.95 [-4.57, 0.68]	*P* = 0.15	*I^2 = ^* 98%
I-FABP	2	19, 28	187	67.93 [3.43, 132.43]	*P* = 0.04	*I^2 = ^* 72%
Fecal calprotectin	2	23, 38	89	31.78 [-138.88, 202.45]	*P* = 0.72	*I^2 = ^* 65%
LBP	2	23, 38	89	0.10 [-0.31, 0.52]	*P* = 0.63	*I^2 = ^* 0%
HRP	2	28, 29	250	-0.54 [-0.60, -0.49]	*P* < 0.00001	*I^2 = ^* 0%
S/E	1	42	25	0.01 [0.00, 0.02]	*P* = 0.01	Not applicable
Mannitol permeability	1	21	60	-0.74 [-0.87, -0.61]	*P* < 0.00001	Not applicable
PEGs	1	19	67	0.11 [-0.95, 1.17]	*P* = 0.84	Not applicable
Saccharose recovery	1	26	28	-0.30 [-0.56, -0.04]	*P* = 0.03	Not applicable
ZO-1	1	39	36	-0.29 [-0.75, 0.17]	*P* = 0.22	Not applicable
NO	1	19	94	214.06 [56.03, 372.09]	*P* = 0.008	Not applicable

L/M, lactulose/mannitol; L/R, lactulose/rhamnose; S/E, sucralose/erythritol; PEGs, polyethylene glycols; D-LA, D-lactic acid; TER, transepithelial electrical resistance; ZO-1, zonula occluden-1; I-FABP, intestinal fatty acid binding protein; NO, nitric oxide; ET, endotoxin; LPS, lipopolysaccharide; LBP, lipopolysaccharide binding protein; HRP, horseradish peroxidase; DAO, diamine oxidase.

**Figure 2:**
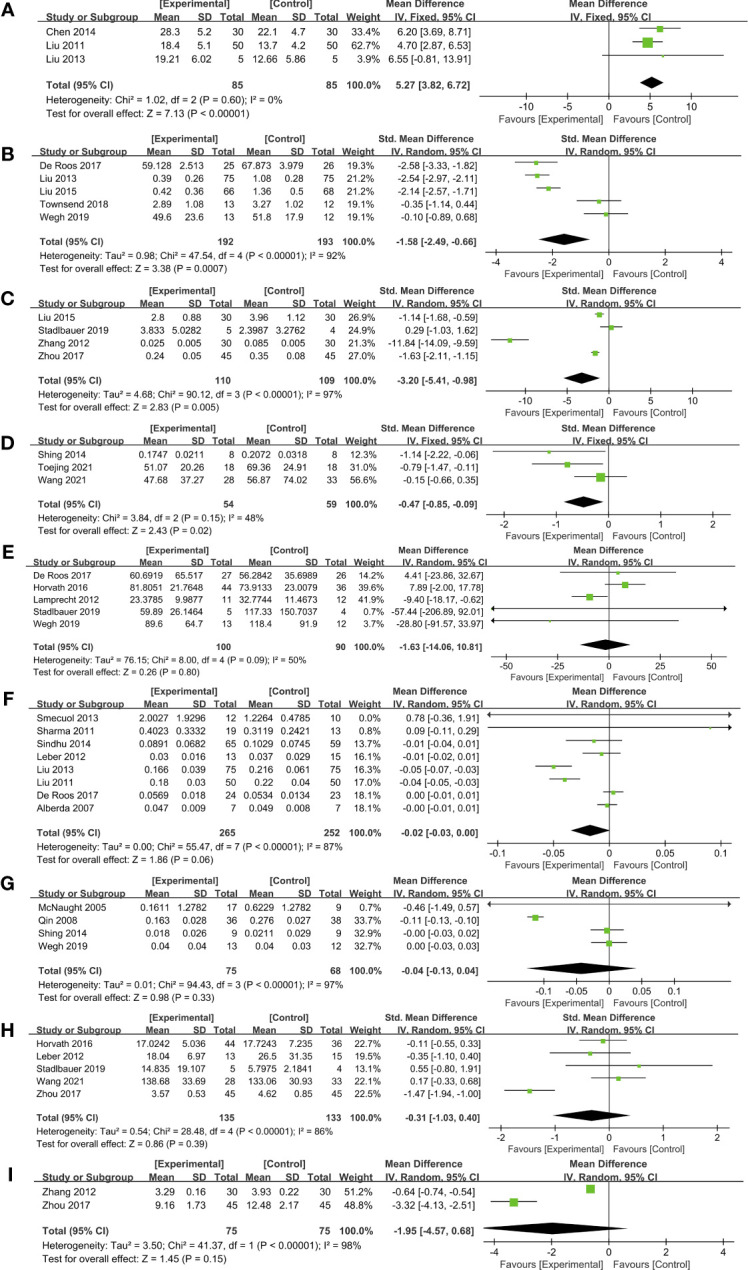
Forest plots of the effect of probiotics on intestinal barrier function. **(A)** TER, **(B)** Serum zonulin, **(C)** ET, **(D)** LPS, **(E)** Fecal zonulin, **(F)** L/M, **(G)** L/R, **(H)** DAO, **(I)** D-LA. DAO, diamine oxidase; D-LA, D-lactic acid; ET, endotoxin; LPS, lipopolysaccharide; L/M, lactulose to mannitol; L/R, lactulose to rhamnose.

A meta-analysis of intestinal barrier function was mainly measured in the TER, serum and fecal zonulin levels, ET, LPS, L/M, L/R, DAO, and D-LA. Three RCTs (21, 28, 29) assessed TER to evaluate the ameliorating effect of probiotics on intestinal barrier function. As indicated by the pooling data (**Figure 2A**), probiotics significantly enhanced the TER compared with placebo (MD, 5.27, 95% CI, 3.82 to 6.72, *P* < 0.00001, *I^2 ^* = 0%).

Measured by serum zonulin concentrations, pooling data from five studies (22, 27, 29, 40, 42) which included 385 subjects demonstrating a significant improvement in probiotic intervention on gut barrier function compared to placebo (SMD, -1.58, 95% CI, -2.49 to -0.66, *P* = 0.0007; **Figure 2B**). Subgroup analysis was conducted based on the country, population characteristics, and duration of treatment (**Table 3**). Among these five RCTs that measured serum zonulin levels, one study (40) included athletes, and the other four (22, 27, 29, 42) were patients. As revealed by the subgroup analysis, RCTs of patients suggested a remarkable decrease in serum zonulin level (SMD, -1.87, 95% CI, -2.77 to -0.98, *P* < 0.0001), while the data from athletes did not exhibit significant changes. In addition to subject type, the intervention duration may also affect the efficacy of probiotics. As revealed in the subgroup analysis (**Table 3**), three RCTs (22, 40, 42) with treatment more extended than four weeks did not achieve significant efficacy. However, two studies (27, 29) involving 284 patients administered probiotics for less than four weeks have notably enhanced the intestinal barrier function assessed by serum zonulin levels (SMD, -2.34, 95% CI, -2.64 to -2.03, *P* < 0.00001) with low statistical heterogeneity (*I^2 ^* = 40%).

**Table 3:** Subgroup analysis of serum zonulin.

Subgroup by		Studies	Participants	SMD (95% CI)	*I^2^*	*P*	*P* for heterogeneity
Country	China	2	284	-2.34[-2.64, -2.03]	40%	<0.00001	0.20
	Netherlands	2	76	-1.34[-3.77, 1.08]	95%	0.28	<0.00001
	USA	1	5	-0.35[-1.14, 0.44]	NA	0.39	NA
Population characteristics	Athletes	1	25	-0.35[-1.14, 0.44]	NA	0.39	NA
	Patients	4	360	-1.87[-2.77, -0.98]	90%	<0.0001	<0.00001
Duration of treatment	< 4 weeks	2	284	-2.34[-2.64, -2.03]	40%	<0.00001	0.20
	≥4 weeks	3	101	-1.01[-2.58, 0.55]	92%	0.20	<0.00001

Five RCTs (22, 23, 25, 38, 42) measured fecal zonulin levels; however, no significant difference was observed between probiotics and placebo groups (**Figure 2E**). A subgroup analysis based on the duration of treatment revealed an interesting phenomenon (**Table 4**). Among the five studies, four interventions involving 110 subjects lasted less than six weeks. Pooling data from these four RCTs (22, 23, 25, 42) demonstrated a remarkable reduction of fecal zonulin levels after probiotics supplementation (MD, -8.69, 95% CI, -16.99 to -0.40, *P* = 0.04, *I^2 ^* = 0%). This outcome is similar to serum zonulin, implying that the duration of probiotic interventions needs to be a concern during clinical application.

**Table 4:** Subgroup analysis of fecal zonulin.

Subgroup by		Studies	Participants	MD (95% CI)	*I^2^*	*P*	*P* for heterogeneity
Country	Austria	3	112	-1.57[-17.76, 14.62]	72%	0.85	0.03
	Netherlands	2	78	-1.19[-26.96, 24.58]	0%	0.93	0.34
Population characteristics	Endurance trained men	1	23	-9.40 [-18.17, -0.62]	NA	0.04	NA
	Patients	4	167	6.48 [-2.74, 15.70]	0%	0.17	0.57
Duration of treatment	< 6 months	4	110	-8.69 [-16.99, -0.40]	0%	0.04	0.65
	≥6 months	1	80	7.89 [-2.00, 17.78]	NA	0.12	NA

Endotoxin and LPS can increase gut permeability and thus cause a leaky gut. Probiotics potently decreased the level of endotoxin (SMD, -3.20, 95% CI, -5.41 to -0.98, *P* = 0.005, *I^2 ^* = 97%) in four studies (27, 38, 44, 45) (**Figure 2C**). Furthermore, compared with placebo, probiotics in three RCTs (34, 39, 41) showed a significant reduction in LPS levels (SMD, -0.47, 95% CI, -0.85 to -0.09, *P* = 0.02) with low heterogeneity (*I^2 ^* = 48%; **Figure 2D**).

Of 26 studies included in the data pooling, eight studies (18, 22, 26, 28, 29, 33, 35, 36) composed of 517 subjects reported L/M levels. The forest plot showed no significant L/M level reduction in probiotics groups compared to control groups (MD, -0.02, 95% CI, -0.03 to 0.00, *P* = 0.06) with high heterogeneity *I^2 ^* = 87% (**Figure 2F**). However, according to a subgroup analysis conducted based on population characteristics (**Table 5**), patients with gastrointestinal diseases in four RCTs (28, 29, 35, 36) displayed a significant reduction of L/M levels (MD, -0.04, 95% CI, -0.06 to 0.02, *P* = 0.0001, *I^2 ^* = 60%), indicating that probiotics may have varying power in improving intestinal permeability of patients with different diseases. Similar to L/M, data on L/R from four studies (30, 31, 34, 42) fail to achieve an effective improvement after probiotic intervention (MD, -0.04, 95% CI, -0.13 to 0.04, *P* = 0.33; **Figure 2G**).

**Table 5:** Subgroup analysis of L/M.

Subgroup by		Studies	Participants	MD (95% CI)	*I^2^*	*P*	*P* for heterogeneity
Country	Austria	1	28	-0.01[-0.02, 0.01]	NA	0.42	NA
	Canada	2	36	-0.00 [-0.01, 0.01]	45%	0.67	0.18
	China	2	250	-0.04[-0.05, -0.03]	0%	<0.00001	0.36
	India	2	156	-0.01 [-0.04, 0.01]	3%	0.34	0.31
	Netherlands	1	47	0.00 [-0.01, 0.01]	NA	0.45	NA
Population characteristics	Critically ill patients	1	14	-0.00 [-0.01, 0.01]	NA	0.66	NA
	Patients with migraine	1	47	0.00 [-0.01, 0.01]	NA	0.45	NA
	Patients with metabolic syndrome	1	28	-0.01 [-0.02, 0.01]	NA	0.42	NA
	Patients with acute pancreatitis	1	32	0.09 [-0.11, 0.29]	NA	0.37	NA
	Patients with gastrointestinal diseases	4	396	-0.04[-0.06, -0.02]	60%	0.0001	0.06
Age	<18	1	124	-0.01 [-0.04, 0.01]	NA	0.28	NA
	≥18	7	393	-0.02 [-0.04, 0.00]	89%	0.09	<0.00001
Duration of treatment	< 2 weeks	2	46	0.00 [-0.01, 0.01]	0%	0.69	0.36
	≥2 weeks	6	471	-0.02 [-0.04, 0.00]	90%	0.07	<0.00001

Five studies (23, 26, 38, 41, 45) reported DAO (SMD, -0.31, 95% CI, -1.03 to 0.40, P=0.39; **Figure 2H**; **Table 6**) and two studies (44, 45) tested D-LA (MD, -1.95, 95% CI, -4.57 to 0.68, *P* = 0.15; **Figure 2I**), however, no significant changes have been observed.

**Table 6:** Subgroup analysis of DAO.

Subgroup by		Studies	Participants	SMD (95% CI)	*I^2^*	*P*	*P* for heterogeneity
Country	Austria	3	117	-0.12[-0.49, 0.24]	0%	0.52	0.52
	China	2	151	-0.65 [-2.26, 0.96]	95%	0.43	<0.00001
Age	<18	1	90	-1.47[-1.94, -1.00]	NA	<0.00001	NA
	≥18	4	178	-0.02 [-0.32, 0.28]	0%	0.89	0.54
Design	Placebo	3	150	0.04 [-0.28, 0.36]	0%	0.80	0.53
	Conventional treatment	2	118	-0.95 [-2.04, 0.15]	84%	0.09	0.01
Duration of treatment	< 2 weeks	2	151	-0.65 [-2.26, 0.96]	95%	0.43	<0.00001
	≥2 weeks	3	117	-0.12 [-0.49, 0.24]	0%	0.52	0.52

### 3.5 Effects of probiotics on inflammation

**Figure 3:**
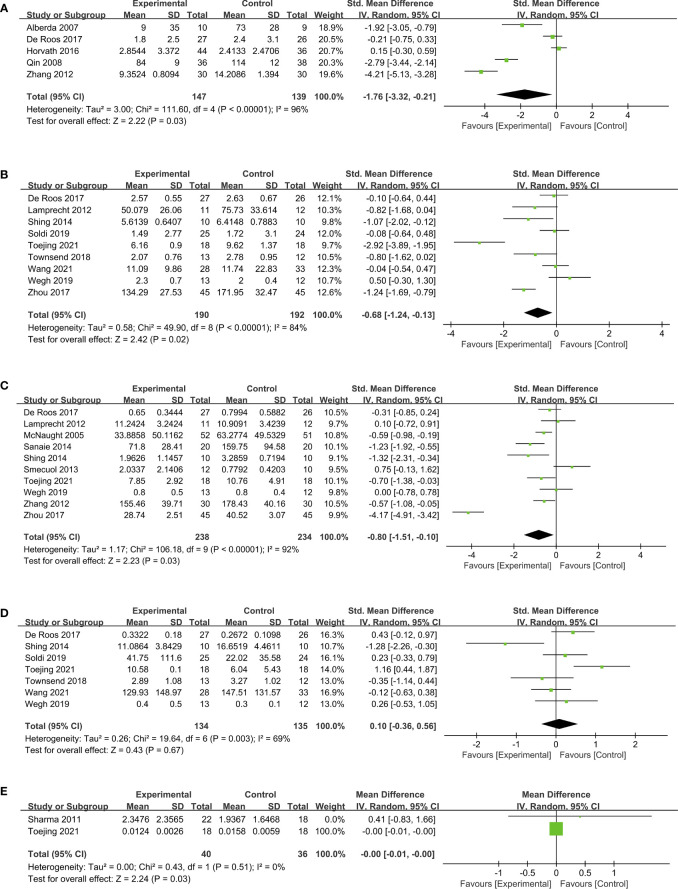
Forest plots of overall pooled data on the effect of probiotics on inflammation. **(A)** CRP, **(B)** TNF-α, **(C)** IL-6, **(D)** IL-10, **(E)** hsCRP. CRP, C-reactive protein; hsCRP, high-sensitivity C-reactive protein; IL-6, interleukin 6; IL-10, interleukin 10; TNF-α: tumor necrosis factor-alpha.

The evaluation of probiotics on inflammation was presented by the levels of CRP, TNF-α, IL-6, IL-10, and hsCRP. The CRP level was measured in five RCTs (18, 22, 23, 31, 44) in 286 patients. Probiotics exhibited dramatically better efficacy over placebo in reducing CRP levels and thus exerting anti-inflammatory activities (SMD, -1.76; 95% CI, -3.32 to -0.21; *P* = 0.03; **Figure 3A**). Subgroup analysis based on the duration of treatment indicated that three studies (18, 31, 44) involving 153 subjects with probiotic intervention less than three months had an even greater reduction of CRP (SMD, -2.99; 95% CI, -4.17 to -1.82; *P* < 0.00001). In contrast, two studies (22, 23) treated with probiotics for more than three months displayed no significant change in CRP levels (**Table S6**). TNF-α levels were revealed by pooled data of nine studies (22, 25, 34, 37, 39–42, 45) involving 382 people, showed a decreasing change in probiotic groups compared to control groups (SMD, -0.68; 95% CI, -1.24 to -0.13; *P* = 0.02; **Figure 3B**). Further detailed subgroup analysis is presented in **Table S7**.

Ten RCTs (22, 25, 30, 32, 34, 36, 39, 42, 44, 45) involving 472 people were pooled to examine the effect of probiotics on IL-6 levels. As indicated in the forest plot (**Figure 3C**), consumption of probiotics induced decreased IL-6 levels (SMD, -0.80; 95% CI, -1.51 to -0.10; *P* = 0.03) with high heterogeneity (*I^2 ^* = 92%). We performed subgroup analyses from various aspects, including countries, population characteristics, age of research subjects, study design, and duration of treatment (**Table S8**). It is worth noting that eight studies (22, 25, 32, 34, 36, 39, 42, 44) applied placebo as control groups had a greater reduction of IL-6 levels than that of conventional treatment (SMD, -0.42; 95% CI, -0.84 to -0.01; *P* = 0.05). In addition, supplementing probiotics for less than three months in six studies (30, 32, 34, 36, 44, 45) had more strength in reducing IL-6 levels compared to those for more than three months (SMD, -1.19; 95% CI, -2.31 to -0.07; *P* = 0.04). However, the probiotic intervention failed to achieve any significant changes in levels of IL-10 (**Figure 3D**; **Table S9**) and hsCRP (**Figure 3E**).

### 3.6 Effects of probiotics on immune function

**Figure 4:**
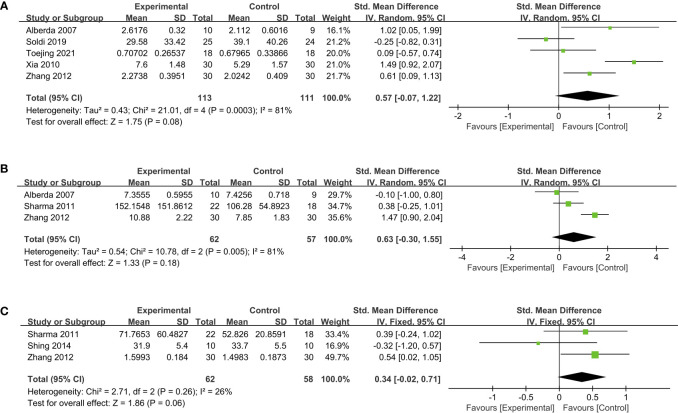
Forest plots of the effect of probiotics on immunoglobulin levels. **(A)** IgA, **(B)** IgG, **(C)** IgM. IgA, Immunoglobulin A; IgG, Immunoglobulin G, IgM, Immunoglobulin M.

The pooled data to evaluate the impact of probiotics on immune function were presented by IgA, IgG, and IgM levels. Compared to placebo groups, supplementing probiotics in five studies (18, 37, 39, 43, 44) did not demonstrate any significant elevation of IgA level (SMD, 0.57; 95% CI, -0.07 to 1.22; *P* = 0.08; **Figure 4A**). In addition, according to meta-analysis, probiotics also failed to effectively improve the levels of IgG (SMD, 0.63; 95% CI, -0.30 to 1.55; *P* = 0.18; **Figure 4B**) and IgM (SMD, 0.34; 95% CI, -0.02 to 0.71; *P* = 0.06; **Figure 4C**) (18, 33, 34, 44).

### 3.7 Effects of probiotics on gut microbiota compositions

**Figure 5:**
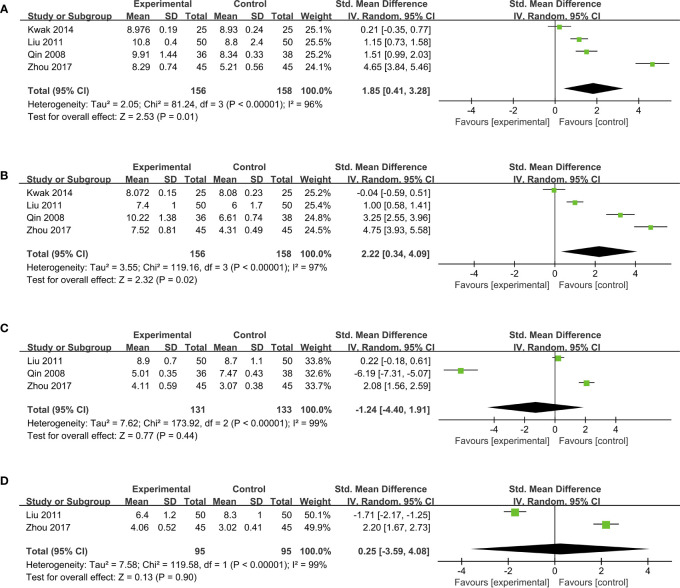
Forest plots of pooled data on the effect of probiotics on microbial abundance. **(A)** *Bifidobacterium*, **(B)** *Lactobacillus*, **(C)** *Enterococcus*, **(D)** *Enterobacteriaceae*.

Probiotics can also modulate the structure of gut microbiota. The data from *Bifidobacterium*, *Lactobacillus*, *Enterococcus*, and *Enterobacteriaceae* were pooled into the meta-analysis. Four studies (24, 28, 31, 45) indicated that, compared to placebo groups, the supplementation of probiotics significantly boosted the enrichment of *Bifidobacterium* (SMD, 1.85, 95% CI, 0.41 to 3.28; *P* = 0.01) in a high heterogeneity (*I^2 ^* = 96%; **Figure 5A**). As for the abundance of *Lactobacillus*, a remarkable increase in probiotic groups was observed from the meta-analysis (SMD, 2.22; 95% CI, 0.34 to 4.09; *P* = 0.02; *I^2 ^* = 97%) after pooling data from four studies (24, 28, 31, 45) (**Figure 5B**). However, no notable difference in *Enterococcus* levels between probiotics and placebo groups was presented in a forest plot containing data from three studies (28, 31, 45) (SMD, -1.24; 95% CI, -4.40 to 1.91; *P* = 0.44; **Figure 5C**). Pooling data from two RCTs (28, 45) also indicated no significant change in the abundance of *Enterobacteriaceae* after probiotic intervention (SMD, 0.25; 95% CI, -3.59 to 4.08; *P* = 0.90; **Figure 5D**).

## 4 Discussion

**Figure 6:**
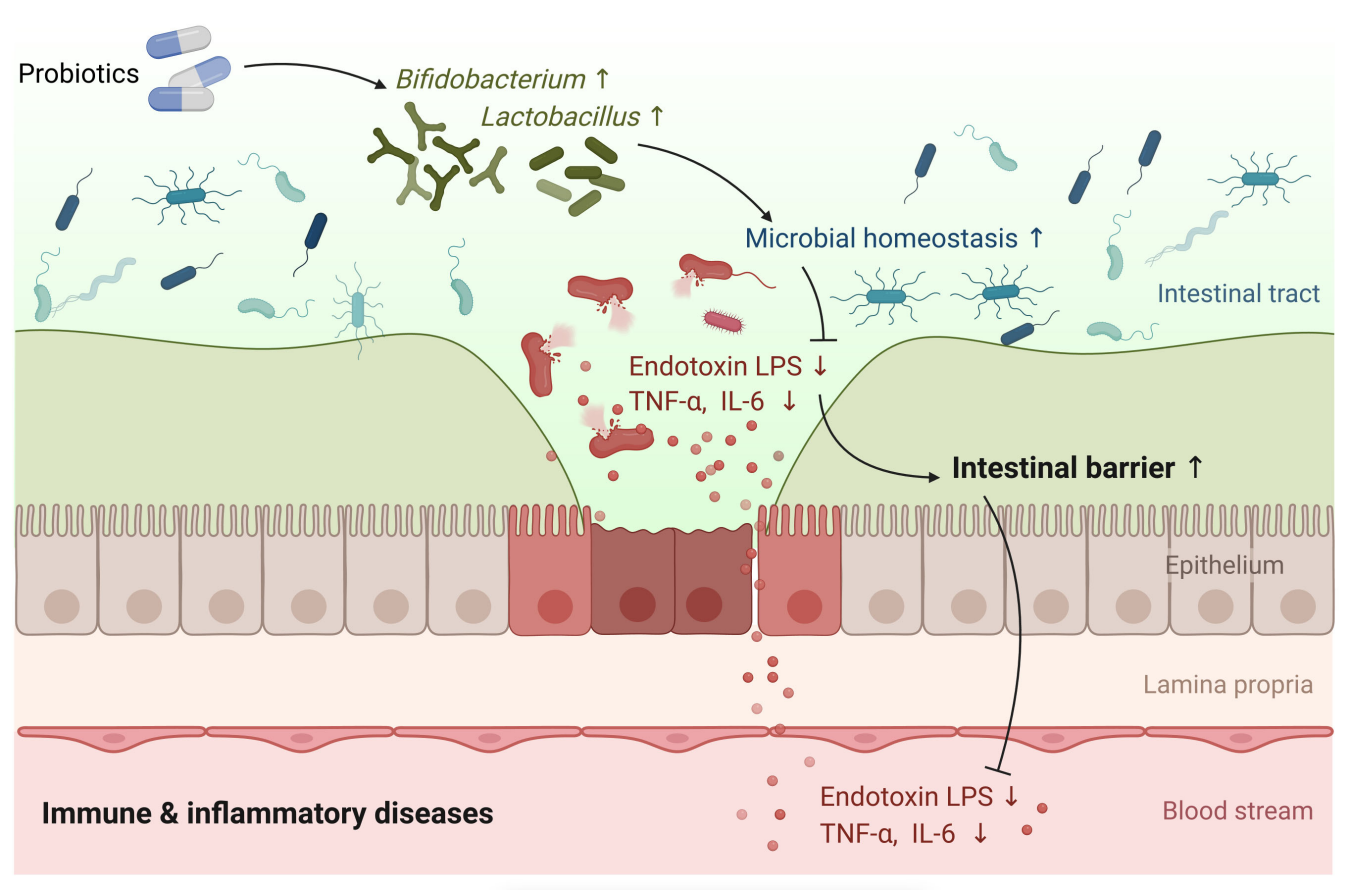
Graphical illustration describes the effect of probiotics in fortifying the intestinal barrier function in immune and inflammation diseases.

The present systematic review and meta-analysis reviewed 28 studies and synthesized 26 studies to assess the effects of single- or multi-strain probiotics. Critical to this study is the fact that probiotics could significantly improve intestinal barrier function according to specific indicators (**Figure 6**). The administration of probiotics significantly stimulated TER and decreased serum zonulin, ET, and LPS levels, as shown in the pooled results. However, we did not observe the effectiveness of probiotics in reducing the levels of fecal zonulin, L/E ratio, L/R ratio, DAO, and D-LA. The meta-analysis also indicated that probiotic supplementation could reduce inflammatory factors such as CRP, IL-6, and TNF-α but did not affect IL-10 and hsCRP. Furthermore, this study also demonstrated that probiotics could modulate gut microbiota compositions by elevating the abundances of *Bifidobacterium* and *Lactobacillus*.

The administration of probiotics decreased the serum zonulin level, indicating a beneficial effect on the intestinal barrier. Zonulin, involved in macromolecule trafficking, is a physiological modulator of intercellular tight junctions, and zonulin level is a hallmark of evaluating intestinal permeability (46, 47). Elevated serum zonulin level is associated with intestinal barrier leakage, microecological dysregulation, and inflammation (42, 48). Fecal zonulin was not significantly correlated with any other markers, which implied that serum zonulin is a better indicator of intestinal permeability than fecal. Subgroup analysis of this study also revealed a significant reduction of fecal zonulin induced by probiotic intervention for less than six weeks. Furthermore, TER is relevant to transepithelial permeability, and increased TER levels of the probiotic group also demonstrated improved intestinal barrier functions.

Probiotics such as *Lactobacillus* and *Bifidobacterium* can down-regulate the proportion of Gram-negative bacteria and reduce the gut-derived LPS production. Inflammatory cytokines, e.g., TNF-α and IL-1β (49, 50), which are crucial intestinal barrier destructors, can also be decreased, therefore protecting the intestinal barrier function (51). Probiotics are also found to modulate the properties of the mucus layer and improve intestinal epithelium permeability (52). A damaged intestinal barrier may lead to increased mucosal permeability and endotoxins entering the circulatory system. The intestinal mucosa with intact physiological function, constitutes a barrier to bacteria and endotoxin, which can stop the gut from leaking toxins and LPS into the blood. Our study proved that the consumption of probiotics could remarkably reduce circulating endotoxin and LPS, demonstrating probiotics’ benefiting role in maintaining gut homeostasis.

The high recovery rates and negligible effects on osmotic pressure in the recipient lumen make Lactulose, mannitol, and rhamnose ideal sugar molecular probes, and their excretion rate ratios (L/M, L/R) are widely accepted indicators to measure intestinal barrier function. In this study, probiotic supplementation did not significantly decrease the L/M ratio. In addition, the L/R ratio also showed no significant differences between probiotic groups and placebo groups.

DAO, a highly active intracellular enzyme in the cytoplasm of intestinal mucosal villous cells, can reflect the maturity and integrity of intestinal epithelial cells and is a sensitive indicator to show the functional status of the intestinal mucosal barrier. In our meta-analysis, five studies measured DAO and failed to achieve an effective reduction of DAO level. D-LA, another primary outcome measure regarding intestinal barrier function, has a significant positive correlation with intestinal mucosal injury scores. Still, there was no evidence to prove that probiotics reduced D-LA levels. However, noteworthy, only two studies were pooled into our analysis, and the forest plot of D-LA showed a high statistical heterogeneity, which suggested that more studies are warranted in the future to accurately determine the impact of probiotics on the gut barrier measured by D-LA.

Regarding the inflammation management of probiotics, we found that the concentrations of CRP, IL-6, and TNF-α dramatically decreased after probiotic treatment. CRP is an acute-phase reactant protein in the plasma, and concentrations increase significantly during acute and chronic inflammation (53). IL-6 is a lymphokine produced by activated T cells and fibroblasts, and TNF-α is the primary proinflammatory cytokine. IL-6 and TNF-α are cytokines that can disrupt the gut barrier integrity and indirectly increase intestinal permeability, thus resulting in bacteria translocation (54), which has been confirmed both *in vivo* and *in vitro* studies (55). Compared to control groups, a significant decrease in these inflammatory factors was observed in probiotic groups. Subgroup analysis further revealed that interventions of less than three months would exhibit better efficacy in reducing the yield of CRP and IL-6. It is believed that probiotics have a significant impact on the mucosal and systemic immune systems by activating multiple immune mechanisms, such as introducing a Th1 profile response with high levels of IL-10 (56). While the levels of IL-10 and immune indicators, such as IgA, IgG, and IgM, had no differences comparing probiotic groups to control groups in our study, indicating that probiotics did not demonstrate any significant improvement in immune functions.

Probiotics may also modulate the compositions of gut microbiota. There are a variety of typical microorganisms in the intestinal tract, and the commensal flora forms a biological barrier by adhering to or binding to the intestinal mucosa. Microorganisms that play an essential role in the intestinal biological barrier are some specific anaerobic bacteria, including *Bifidobacterium* and *Lactobacillus*. These specialized anaerobic bacteria tightly bind to the intestinal epithelium through adhesion and form a pellicle barrier, which can compete to inhibit the binding of pathogenic bacteria to the intestinal epithelium and inhibit their colonization and growth. Furthermore, our study found that intervention with probiotics increased the abundance of *Bifidobacterium* and *Lactobacillus*, which is beneficial for hindering a large amount of endotoxin from entering the circulatory system and preventing triggering functional damage to various organ systems (57).

We tried to assess the role of probiotics in improving intestinal barrier function comprehensively from various perspectives. In addition, subgroup meta-analyses were conducted according to multiple aspects, e.g., country, population characteristics, and study design. However, the limitations of the present study cannot be ignored when interpreting and extrapolating our findings. First, the heterogeneity of some analyses remained high even though a subgroup meta-analysis was performed, which may influence the accuracy of the results. Second, the type or other specific probiotics information in some studies was unclear, which limited further analysis. Furthermore, the included studies for meta-analysis measuring gut barriers involved a wide range of indicators, and different methods may lead to different results; therefore, more comprehensive studies will be warranted in the future to draw definitive conclusions about the role of probiotics on intestinal barrier function.

These findings suggested that probiotics could improve intestinal barrier function to some extent, but more high-quality RCTs are needed to achieve a solid conclusion. In addition, further in-depth research is required to target the precise dose, intervention duration, and strains of probiotics to provide valuable instructions for clinical practice.

## Data availability statement

The original contributions presented in the study are included in the article/**Supplementary Material**. Further inquiries can be directed to the corresponding authors.

## Author contributions

J-YW, HY, and C-SY conceived the idea for the paper. YZ, PT, ZZ, and YW conducted the literature searching, and meta-analysis. YZ, PT, ZZ, and HY conducted the quality assessment of included studies. YZ, PT, ZZ, AZ, J-YW, HY drafted the manuscript. C-ZW, C-SY contributed data analysis and revised the manuscript. All authors provided critical reviews and approved the final version of the manuscript. All authors contributed to the article and approved the submitted version.

## Supplementary material

### Abbreviations

CENTRAL, Cochrane Central Register of Controlled Trials; CI, confidence interval; CRP, C-reactive protein; D-LA, D-lactic acid; DAO, diamine oxidase; ET, endotoxin; GI, gastrointestinal; hsCRP, high-sensitivity C-reactive protein; I-FABP: intestinal fatty acid-binding protein; IL-6: interleukin 6; IL-10, interleukin 10; IgA, Immunoglobulin A; IgG, Immunoglobulin G; IgM, Immunoglobulin M; L/M, lactulose to mannitol; L/R, lactulose to rhamnose; LPS, lipopolysaccharide; MD, mean difference; RCT, randomized controlled trial; SD, standard deviation; SMD, standard mean difference; TER, transepithelial resistance; TNF-α, tumor necrosis factor-alpha.
