# Supplementary material for: Probiotics fortify intestinal barrier function: a systematic review and meta-analysis of randomized trials
Source: Front Immunol. 2023 Apr 24;14:1143548. doi: 10.3389/fimmu.2023.1143548 (PMC10165082; doi:10.3389/fimmu.2023.1143548)
Supplement: Supplementary file 1 [file DataSheet_1.docx]

**Supplementary Information**

**Probiotics Fortify Intestinal Barrier Function: A Systematic Review and Meta-Analysis of Randomized Trials**

Yanfei Zheng^1,2#^, Zengliang Zhang^3#^, Ping Tang^1,2#^, Yuqi Wu^1,2^, Anqi Zhang^2^, Delong Li^1,2^, Chong-Zhi Wang^4,5^, Jin-Yi Wan^1,2*^, Haiqiang Yao^1,2*^, Chun-Su Yuan^4,5^

**Corresponding authors:**

Jin-Yi Wan, Ph.D., Beijing University of Chinese Medicine, No. 11 N. 3rd Ring Road East, Beijing 100029, China. E-mail: [wanjinyi1128@163.com](mailto:wanjinyi1128@163.com);

Haiqiang Yao, M.D., Ph.D., Beijing University of Chinese Medicine, No. 11 N. 3rd Ring Road East, Beijing 100029, China. E-mail: [haiqiangyao@outlook.com](mailto:haiqiangyao@outlook.com).

^#^ These authors contributed equally to this work.

This supplementary file contains: Figure S1-S2, Table S1-S9.

**Figure S1**. Risk of bias graph.

**
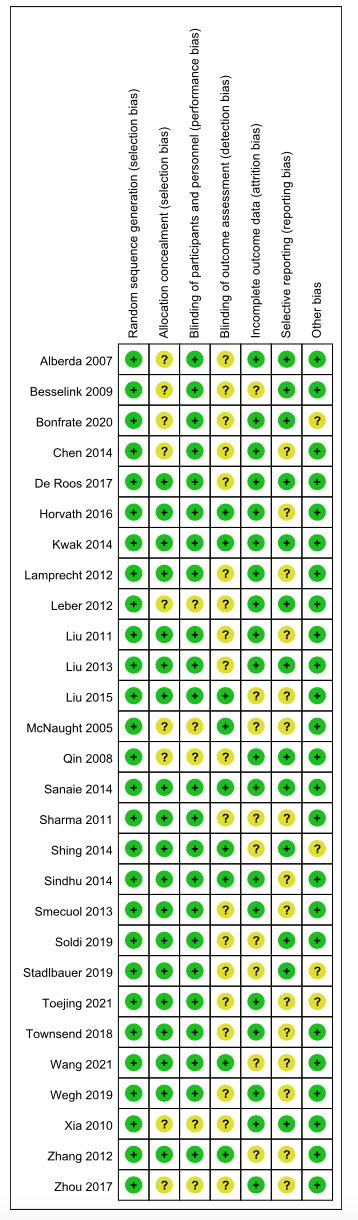
**

**Figure S2.** Risk of bias summary for included studies.

**Table S1.** The PRISMA 2020 checklist

| **Section and Topic** | **Item #** | **Checklist item** | **Location where item is reported** |
| --- | --- | --- | --- |
| **TITLE** | | |  |
| Title | 1 | Identify the report as a systematic review. | Page 1, Title |
| **ABSTRACT** | | |  |
| Abstract | 2 | See the PRISMA 2020 for Abstracts checklist. | Page 2, Abstract |
| **INTRODUCTION** | | |  |
| Rationale | 3 | Describe the rationale for the review in the context of existing knowledge. | Page 4, Introduction |
| Objectives | 4 | Provide an explicit statement of the objective(s) or question(s) the review addresses. | Page 4-5, Introduction |
| **METHODS** | | |  |
| Eligibility criteria | 5 | Specify the inclusion and exclusion criteria for the review and how studies were grouped for the syntheses. | Page 5, Methods |
| Information sources | 6 | Specify all databases, registers, websites, organisations, reference lists and other sources searched or consulted to identify studies. Specify the date when each source was last searched or consulted. | Page 5, Methods |
| Search strategy | 7 | Present the full search strategies for all databases, registers and websites, including any filters and limits used. | Supplementary Table S2-S5 |
| Selection process | 8 | Specify the methods used to decide whether a study met the inclusion criteria of the review, including how many reviewers screened each record and each report retrieved, whether they worked independently, and if applicable, details of automation tools used in the process. | Page 5, Methods |
| Data collection process | 9 | Specify the methods used to collect data from reports, including how many reviewers collected data from each report, whether they worked independently, any processes for obtaining or confirming data from study investigators, and if applicable, details of automation tools used in the process. | Page 5-6, Methods |
| Data items | 10a | List and define all outcomes for which data were sought. Specify whether all results that were compatible with each outcome domain in each study were sought (e.g. for all measures, time points, analyses), and if not, the methods used to decide which results to collect. | Page 6-7, Methods; Table 1 |
|  | 10b | List and define all other variables for which data were sought (e.g. participant and intervention characteristics, funding sources). Describe any assumptions made about any missing or unclear information. | Page 6-7, Methods; Table 1 |
| Study risk of bias assessment | 11 | Specify the methods used to assess risk of bias in the included studies, including details of the tool(s) used, how many reviewers assessed each study and whether they worked independently, and if applicable, details of automation tools used in the process. | Page 6, Methods |
| Effect measures | 12 | Specify for each outcome the effect measure(s) (e.g. risk ratio, mean difference) used in the synthesis or presentation of results. | Page 7, Methods |
| Synthesis methods | 13a | Describe the processes used to decide which studies were eligible for each synthesis (e.g. tabulating the study intervention characteristics and comparing against the planned groups for each synthesis (item #5)). | Page 6-7, Methods |
|  | 13b | Describe any methods required to prepare the data for presentation or synthesis, such as handling of missing summary statistics, or data conversions. | Page 6-7, Methods |
|  | 13c | Describe any methods used to tabulate or visually display results of individual studies and syntheses. | Page 6-7, Methods |
|  | 13d | Describe any methods used to synthesize results and provide a rationale for the choice(s). If meta-analysis was performed, describe the model(s), method(s) to identify the presence and extent of statistical heterogeneity, and software package(s) used. | Page 6-7, Methods |
|  | 13e | Describe any methods used to explore possible causes of heterogeneity among study results (e.g. subgroup analysis, meta-regression). | Page 6-7, Methods |
|  | 13f | Describe any sensitivity analyses conducted to assess robustness of the synthesized results. | Page 6-7, Methods |
| Reporting bias assessment | 14 | Describe any methods used to assess risk of bias due to missing results in a synthesis (arising from reporting biases). | Page 6-7, Methods |
| Certainty assessment | 15 | Describe any methods used to assess certainty (or confidence) in the body of evidence for an outcome. | Page 6-7, Methods |
| **RESULTS** | | |  |
| Study selection | 16a | Describe the results of the search and selection process, from the number of records identified in the search to the number of studies included in the review, ideally using a flow diagram. | Page 7-8, Results |
|  | 16b | Cite studies that might appear to meet the inclusion criteria, but which were excluded, and explain why they were excluded. | Page 7-8, Results |
| Study characteristics | 17 | Cite each included study and present its characteristics. | Page 8-9, Results; Table 1 |
| Risk of bias in studies | 18 | Present assessments of risk of bias for each included study. | Page 9, Results; Supplementary Figure S1-S2 |
| Results of individual studies | 19 | For all outcomes, present, for each study: (a) summary statistics for each group (where appropriate) and (b) an effect estimate and its precision (e.g. confidence/credible interval), ideally using structured tables or plots. | Page 9-13, Results; Figure 2-5 |
| Results of syntheses | 20a | For each synthesis, briefly summarise the characteristics and risk of bias among contributing studies. | Page 9-13, Results; Figure 2-5 |
|  | 20b | Present results of all statistical syntheses conducted. If meta-analysis was done, present for each the summary estimate and its precision (e.g. confidence/credible interval) and measures of statistical heterogeneity. If comparing groups, describe the direction of the effect. | Page 9-13, Results; Figure 2-5; Table 3-6; Supplementary Table 6-9 |
|  | 20c | Present results of all investigations of possible causes of heterogeneity among study results. | Page 9-13, Results; Figure 2-5; Table 3-6; Supplementary Table 6-9 |
|  | 20d | Present results of all sensitivity analyses conducted to assess the robustness of the synthesized results. | Page 9-13, Results |
| Reporting biases | 21 | Present assessments of risk of bias due to missing results (arising from reporting biases) for each synthesis assessed. | Page 9-13, Results |
| Certainty of evidence | 22 | Present assessments of certainty (or confidence) in the body of evidence for each outcome assessed. | Page 9-13, Results |
| **DISCUSSION** | | |  |
| Discussion | 23a | Provide a general interpretation of the results in the context of other evidence. | Page 13, Discussion |
|  | 23b | Discuss any limitations of the evidence included in the review. | Page 16, Discussion |
|  | 23c | Discuss any limitations of the review processes used. | Page 16, Discussion |
|  | 23d | Discuss implications of the results for practice, policy, and future research. | Page 15-16, Discussion |
| **OTHER INFORMATION** | | |  |
| Registration and protocol | 24a | Provide registration information for the review, including register name and registration number, or state that the review was not registered. | Page 5, Methods |
|  | 24b | Indicate where the review protocol can be accessed, or state that a protocol was not prepared. | Page 5, Methods |
|  | 24c | Describe and explain any amendments to information provided at registration or in the protocol. | Page 5, Methods |
| Support | 25 | Describe sources of financial or non-financial support for the review, and the role of the funders or sponsors in the review. | Page 16, Acknowledgments |
| Competing interests | 26 | Declare any competing interests of review authors. | Page 17, Declaration of Competing Interest |
| Availability of data, code and other materials | 27 | Report which of the following are publicly available and where they can be found: template data collection forms; data extracted from included studies; data used for all analyses; analytic code; any other materials used in the review. | Page 17, Data availability |

*From:*  Page MJ, McKenzie JE, Bossuyt PM, Boutron I, Hoffmann TC, Mulrow CD, et al. The PRISMA 2020 statement: an updated guideline for reporting systematic reviews. BMJ 2021;372:n71. doi: 10.1136/bmj.n71

**Table S2.** Search strategy of PubMed

| # | Searches |
| --- | --- |
| 1 | (probioti*[Title/Abstract]) OR (probiotics[MeSH Terms]) |
| 2 | (((((((((((Lactobacillus[Title/Abstract]) OR (Bifidobacterium[Title/Abstract])) OR (Enterococcus[Title/Abstract]) OR Saccharomyces boulardii[Title/Abstract]) OR (Streptococcus[Title/Abstract])) OR (Saccharomyces[Title/Abstract])) OR (Bacillus[Title/Abstract])) OR (Lactococcus[Title/Abstract])) OR (Leuconostoc[Title/Abstract])) OR (Propionibacterium[Title/Abstract])) OR (Pediococcus[Title/Abstract])) OR (Staphylococcus[Title/Abstract])) OR (Kluyveromyces[Title/Abstract]) |
| 3 | ((((((((gut integrity[Title/Abstract]) OR (intestinal integrity[Title/Abstract])) OR (gut permeability[Title/Abstract])) OR (intestinal permeability[Title/Abstract])) OR ((((gut barrier[Title/Abstract]) OR (intestinal barrier[Title/Abstract])) OR (mucus barrier[Title/Abstract])) OR (mucosal barrier[Title/Abstract]))) OR (epiethelial barrier[Title/Abstract])) OR (leaky gut[Title/Abstract])) OR (barrier integrity[Title/Abstract])) OR (epiethelial integrity[Title/Abstract]) |
| 4 | #1 OR #2 |
| 5 | #3 AND #4 |
| 6 | ((((((((((((randomized controlled trial[Publication Type]) OR (controlled clinical trial[Publication Type])) OR (clinical trials as topic[MeSH Terms])) OR (random allocation[MeSH Terms])) OR (randomized controlled trials as topic[MeSH Terms])) OR (clinical trial[Publication Type])) OR (clinical trial[Text Word])) OR (singl*[Text Word])) OR (doubl*[Text Word])) OR (trebl*[Text Word])) OR (trepl*[Text Word])) OR (randomly[Text Word])) OR (trial[Text Word]) |
| 7 | #5 AND #6 |

**Table S3.** Search strategy of Web of Science: Science Citation Index Expanded

| # | Searches |
| --- | --- |
| 1 | TS=(probioti*) |
| 2 | ((((((((((((TS=(Lactobacillus)) OR TS=(Bifidobacterium)) OR TS=(Enterococcus)) OR TS=(Saccharomyces boulardii)) OR TS=(Streptococcus)) OR TS=(Saccharomyces)) OR TS=(Bacillus)) OR TS=(Lactococcus)) OR TS=(Leuconostoc)) OR TS=(Propionibacterium)) OR TS=(Pediococcus)) OR TS=(Staphylococcus)) OR TS=(Kluyveromyces) |
| 3 | #1 OR #2 |
| 4 | (((((((((((TS=(gut integrity)) OR TS=(intestinal integrity)) OR TS=(gut permeability)) OR TS=(intestinal permeability)) OR TS=(gut barrier)) OR TS=(intestinal barrier)) OR TS=(mucus barrier)) OR TS=(mucosal barrier)) OR TS=(epithelial barrier)) OR TS=(epithelial integrity)) OR TS=(barrier integrity)) OR TS=(leaky gut) |
| 5 | (((((((((TS=(randomized controlled tria*)) OR TS=(controlled clinical tria*)) OR TS=(singl*)) OR TS=(doubl*)) OR TS=(trebl*)) OR TS=(trepl*)) OR TS=(placeb*)) OR TS=(random*)) OR TS=(clinical tria*)) OR TS=(tria*) |
| 6 | #3 AND #4 AND #5 |

**Table S4.** Search strategy of Cochrane Central Register of Controlled Trials (CENTRAL)

| # | Searches |
| --- | --- |
| 1 | (probioti*):tiab,kw |
| 2 | MeSH descriptor: [Probiotics] explode all trees |
| 3 | (“Lactobacillus”):ti,ab,kw OR (“Bifidobacterium”):ti,ab,kw OR (“Enterococcus”):ti,ab,kw OR (“Saccharomyces boulardii”):ti,ab,kw OR (“Streptococcus”):ti,ab,kw |
| 4 | (“Saccharomyces”):ti,ab,kw OR (“Bacillus”):ti,ab,kw OR (“Lactococcus”):ti,ab,kw OR (“Leuconostoc”):ti,ab,kw OR (“Propionibacterium”):ti,ab,kw |
| 5 | (“Pediococcus”):ti,ab,kw OR (“Staphylococcus”):ti,ab,kw OR (“Kluyveromyces”):ti,ab,kw |
| 6 | #1 OR #2 OR #3 OR #4 OR #5 |
| 7 | (gut integrity):ti,ab,kw OR (intestinal integrity):ti,ab,kw OR (gut permeability):ti,ab,kw OR (“intestinal permeability”):ti,ab,kw |
| 8 | (gut barrier):ti,ab,kw OR (intestinal barrier):ti,ab,kw OR (mucus barrier):ti,ab,kw OR (mucosal barrier):ti,ab,kw OR (epithelial barrier):ti,ab,kw |
| 9 | (leaky gut):ti,ab,kw OR (barrier integrity):ti,ab,kw OR (epithelial integrity):ti,ab,kw |
| 10 | #7 OR #8 OR #9 |
| 11 | #6 AND #10 |

**Table S5.** Search strategy of Embase

| [#](http://ovidsp.tx.ovid.com/sp-3.13.1a/ovidweb.cgi?&S=PCMGFPEFFNDDILLNNCLKAALBDCMDAA00&Sort+Sets=descending) | Searches |
| --- | --- |
| 1 | 'probiotic agent'/exp |
| 2 | probioti*:ti,ab,kw |
| 3 | #1 OR #2 |
| 4 | 'lactobacillus'/exp OR 'bifidobacterium'/exp OR 'enterococcus'/exp OR 'saccharomyces boulardii'/exp OR 'streptococcus'/exp OR 'saccharomyces'/exp OR 'bacillus'/exp OR 'lactococcus'/exp OR 'leuconostoc'/exp OR 'propionibacterium'/exp OR 'pediococcus'/exp OR 'staphylococcus'/exp OR 'kluyveromyces'/exp |
| 5 | lactobacillus:ti,ab,kw OR bifidobacterium:ti,ab,kw OR enterococcus:ti,ab,kw OR 'saccharomyces boulardii':ti,ab,kw OR streptococcus:ti,ab,kw OR saccharomyces:ti,ab,kw OR bacillus:ti,ab,kw OR lactococcus:ti,ab,kw OR leuconostoc:ti,ab,kw OR propionibacterium:ti,ab,kw OR pediococcus:ti,ab,kw OR staphylococcus:ti,ab,kw OR kluyveromyces:ti,ab,kw |
| 6 | #3 OR #4 OR #5 |
| 7 | 'gut integrity':ti,ab,kw OR 'intestinal integrity':ti,ab,kw OR 'gut permeability':ti,ab,kw OR 'intestinal permeability':ti,ab,kw OR 'gut barrier':ti,ab,kw OR 'intestinal barrier':ti,ab,kw OR 'mucus barrier':ti,ab,kw OR 'mucosal barrier':ti,ab,kw OR 'epithelial barrier':ti,ab,kw OR 'leaky gut':ti,ab,kw OR 'barrier integrity':ti,ab,kw OR 'epithelial integrity':ti,ab,kw |
| 8 | #6 AND #7 |
| 9 | 'randomized controlled trial (topic)'/exp OR 'randomization'/exp OR 'single blind procedure'/exp OR 'double blind procedure'/exp OR 'placebo'/exp OR 'clinical trial (topic)'/exp OR 'controlled clinical trial (topic)'/exp |
| 10 | 'randomized controlled trial':ti,ab,kw OR 'controlled clinical trial':ti,ab,kw OR singl*:ti,ab,kw OR doubl*:ti,ab,kw OR trebl*:ti,ab,kw OR trepl*:ti,ab,kw OR randomly:ti,ab,kw OR tria*:ti,ab,kw |
| 11 | #9 OR #10 |
| 12 | #8 AND #11 |

**Table S6.** Subgroup analysis of CRP

| **Subgroup by** | | **Studies** | | **Participants** | | **SMD (95% CI)** | ***I^2^*** | ***P*** | ***P* for heterogeneity** | |
| --- | --- | --- | --- | --- | --- | --- | --- | --- | --- | --- |
| Country | Austria | | 1 | | 80 | 0.15 [-0.30, 0.59] | NA | 0.52 | | NA |
|  | Canada | | 1 | | 19 | -1.92 [-3.05, -0.79] | NA | 0.0009 | | NA |
|  | China | | 2 | | 134 | -3.46 [-4.84, -2.07] | 83% | ＜0.00001 | | 0.01 |
|  | Netherlands | | 1 | | 53 | -0.21 [-0.75, 0.33] | NA | 0.45 | | NA |
| Design | Placebo | | 4 | | 212 | -1.50 [-3.20, 0.20] | 96% | 0.08 | | ＜0.00001 |
|  | Parenteral nutrition | | 1 | | 74 | -2.79 [-3.44, -2.14] | NA | ＜0.00001 | | NA |
| Duration of treatment | < 3 months | | 3 | | 153 | -2.99 [-4.17, -1.82] | 81% | ＜0.00001 | | 0.006 |
|  | ≥3 months | | 2 | | 133 | 0.00 [-0.34, 0.34] | 0% | 0.99 | | 0.32 |

**Table S7.** Subgroup analysis of TNF-α

| **Subgroup by** | | **Studies** | | **Participants** | | **SMD (95% CI)** | ***I^2^*** | ***P*** | ***P* for heterogeneity** | |
| --- | --- | --- | --- | --- | --- | --- | --- | --- | --- | --- |
| Country | Australia | | 1 | | 20 | -1.07[-2.02, -0.12] | NA | 0.03 | | NA |
|  | Austria | | 1 | | 23 | -0.82 [-1.68, 0.04] | NA | 0.06 | | NA |
|  | China | | 2 | | 151 | -0.64 [-1.82, 0.54] | 92% | 0.29 | | 0.0005 |
|  | Italy | | 1 | | 49 | -0.08 [-0.64, 0.48] | NA | 0.79 | | NA |
|  | Netherlands | | 2 | | 78 | 0.09 [-0.36, 0.54] | 33% | 0.69 | | 0.22 |
|  | Thailand | | 1 | | 36 | -2.92 [-3.89, -1.95] | NA | < 0.00001 | | NA |
|  | USA | | 1 | | 25 | -0.80 [-1.62, 0.02] | NA | 0.06 | | NA |
| Population characteristics | Critically ill patients | | 1 | | 61 | -0.04 [-0.54, 0.47] | NA | 0.89 | | NA |
|  | Patients with migraine | | 1 | | 53 | -0.10 [-0.64, 0.44] | NA | 0.73 | | NA |
|  | Patients with type 2 diabetes mellitus | | 1 | | 36 | -2.92 [-3.89, -1.95] | NA | < 0.00001 | | NA |
|  | Athletes | | 3 | | 68 | -0.88 [-1.39, -0.38] | 0% | 0.0006 | | 0.90 |
|  | Healthy adults who self-reported psychological stress | | 1 | | 49 | -0.08 [-0.64, 0.48] | NA | 0.79 | | NA |
|  | Patients with gastrointestinal diseases | | 2 | | 115 | -0.40 [-2.11, 1.31] | 93% | 0.65 | | 0.0002 |
| Age | <18 | | 1 | | 90 | -1.24 [-1.69, -0.79] | NA | ＜0.00001 | | NA |
|  | ≥18 | | 8 | | 292 | -0.60 [-1.19, -0.01] | 82% | 0.05 | | ＜0.00001 |
| Design | Placebo | | 8 | | 292 | -0.60 [-1.19, -0.01] | 82% | 0.05 | | ＜0.00001 |
|  | Conventional treatment | | 1 | | 90 | -1.24 [-1.69, -0.79] | NA | ＜0.00001 | | NA |
| Duration of treatment | < 3 months | | 4 | | 220 | -0.58 [-1.27, 0.10] | 82% | 0.10 | | 0.0008 |
|  | ≥3 months | | 5 | | 162 | -0.79 [-1.79, 0.21] | 88% | 0.12 | | ＜0.00001 |

**Table S8.** Subgroup analysis of IL-6

| **Subgroup by** | | **Studies** | | **Participants** | | **SMD (95% CI)** | ***I^2^*** | ***P*** | ***P* for heterogeneity** | |
| --- | --- | --- | --- | --- | --- | --- | --- | --- | --- | --- |
| Country | Austria | | 1 | | 23 | 0.10 [-0.72, 0.91] | NA | 0.82 | | NA |
|  | Australia | | 1 | | 20 | -1.32[-2.31, -.34] | NA | 0.009 | | NA |
|  | Canada | | 1 | | 22 | 0.75 [-0.13, 1.62] | NA | 0.09 | | NA |
|  | China | | 2 | | 150 | -2.36 [-5.88, 1.17] | 98% | 0.19 | | ＜0.00001 |
|  | Iran | | 1 | | 40 | -1.23[-1.92, -0.55] | NA | 0.0004 | | NA |
|  | Netherlands | | 2 | | 78 | -0.21 [-0.65, 0.24] | 0% | 0.36 | | 0.53 |
|  | Thailand | | 1 | | 36 | -0.70[-1.38, -0.03] | NA | 0.04 | | NA |
|  | UK | | 1 | | 103 | -0.59[-0.98, -0.19] | NA | 0.004 | | NA |
| Population characteristics | Critically ill patients | | 2 | | 143 | -0.85[-1.47, -0.22] | 62% | 0.008 | | 0.11 |
|  | Patients with migraine | | 1 | | 53 | -0.31 [-0.85, 0.24] | NA | 0.27 | | NA |
|  | Patients with type 2 diabetes mellitus | | 1 | | 36 | -0.70[-1.38, -0.03] | NA | 0.04 | | NA |
|  | Athletes | | 2 | | 43 | -0.59 [-1.98, 0.81] | 79% | 0.41 | | 0.03 |
|  | Patients with gastrointestinal diseases | | 4 | | 197 | -1.00 [-2.99, 0.99] | 97% | 0.32 | | ＜0.00001 |
| Age | <18 | | 1 | | 90 | -4.17[-4.91, -3.42] | NA | ＜0.00001 | | NA |
|  | ≥18 | | 9 | | 382 | -0.45 [-0.79, -0.11] | 59% | 0.01 | | 0.01 |
| Design | Placebo | | 8 | | 279 | -0.42[-0.84, -0.01] | 64% | 0.05 | | 0.007 |
|  | Conventional treatment | | 2 | | 193 | -2.36 [-5.87, 1.15] | 99% | 0.19 | | ＜0.00001 |
| Duration of treatment | < 3 months | | 6 | | 335 | -1.19[-2.31, -0.07] | 95% | 0.04 | | ＜0.00001 |
|  | ≥3 months | | 4 | | 137 | -0.28 [-0.62, 0.06] | 0% | 0.10 | | 0.42 |

**Table S9.** Subgroup analysis of IL-10

| **Subgroup by** | | **Studies** | | **Participants** | | **SMD (95% CI)** | ***I^2^*** | ***P*** | ***P* for heterogeneity** | |
| --- | --- | --- | --- | --- | --- | --- | --- | --- | --- | --- |
| Country | Australia | | 1 | | 20 | -1.28[-2.26, 0.30] | NA | 0.01 | | NA |
|  | China | | 1 | | 61 | -0.12 [-0.63, 0.38] | NA | 0.63 | | NA |
|  | Italy | | 1 | | 49 | 0.23 [-0.33, 0.79] | NA | 0.42 | | NA |
|  | Netherlands | | 2 | | 78 | 0.37 [-0.07, 0.82] | 0% | 0.10 | | 0.74 |
|  | Thailand | | 1 | | 36 | 1.16 [0.44, 1.87] | NA | 0.001 | | NA |
|  | USA | | 1 | | 25 | -0.35 [-1.14, 0.44] | NA | 0.39 | | NA |
| Population characteristics | Critically ill patients | | 1 | | 61 | -0.12 [-0.63, 0.38] | NA | 0.63 | | NA |
|  | Patients with migraine | | 1 | | 53 | 0.43 [-0.12, 0.97] | NA | 0.12 | | NA |
|  | Patients with type 2 diabetes mellitus | | 1 | | 36 | 1.16 [0.44, 1.87] | NA | 0.001 | | NA |
|  | Athletes | | 2 | | 45 | -0.77 [-1.68, 0.14] | 52% | 0.10 | | 0.15 |
|  | Healthy adults who self-reported psychological stress | | 1 | | 49 | 0.23 [-0.33, 0.79] | NA | 0.42 | | NA |
|  | Patients with ulcerative colitis | | 1 | | 25 | 0.26 [-0.53, 1.05] | NA | 0.51 | | NA |
| Duration of treatment | < 3 months | | 3 | | 130 | -0.28 [-0.97, 0.42] | 71% | 0.43 | | 0.03 |
|  | ≥3 months | | 4 | | 139 | 0.39 [-0.17, 0.96] | 62% | 0.17 | | 0.05 |
